# Electrospun Nanofibers for Dura Mater Regeneration: A Mini Review on Current Progress

**DOI:** 10.3390/pharmaceutics15051347

**Published:** 2023-04-27

**Authors:** Bishweshwar Pant, Mira Park, Allison A. Kim

**Affiliations:** 1 Carbon Composite Energy Nanomaterials Research Center, Woosuk University, Wanju 55338, Republic of Korea; bisup@woosuk.ac.kr; 2 Woosuk Institute of Smart Convergence Life Care (WSCLC), Woosuk University, Wanju 55338, Republic of Korea; 3 Department of Automotive Engineering, Woosuk University, Wanju 55338, Republic of Korea; 4 Department of Healthcare Management, Woosong University, Daejon 34606, Republic of Korea

**Keywords:** electrospinning, nanofibers, biomedical applications, dura mater repair

## Abstract

Dural defects are a common problem in neurosurgical procedures and should be repaired to avoid complications such as cerebrospinal fluid leakage, brain swelling, epilepsy, intracranial infection, and so on. Various types of dural substitutes have been prepared and used for the treatment of dural defects. In recent years, electrospun nanofibers have been applied for various biomedical applications, including dural regeneration, due to their interesting properties such as a large surface area to volume ratio, porosity, superior mechanical properties, ease of surface modification, and, most importantly, similarity with the extracellular matrix (ECM). Despite continuous efforts, the development of suitable dura mater substrates has had limited success. This review summarizes the investigation and development of electrospun nanofibers with particular emphasis on dura mater regeneration. The objective of this mini-review article is to give readers a quick overview of the recent advances in electrospinning for dura mater repair.

## 1. Electrospinning: History, Setup, and Principle

Electrospinning technology, as an excellent method for producing nano-to-micro-scale fibers, has attracted much attention due to its versatility, rapidness, controllability, low cost, and large surface area to volume ratio micro/nanofibers with uniform or special microscopic morphology [1,2,3]. Electrospinning has had a century-long development [2,4,5]. In 1745, Bose determined that a large electric potential is necessary to generate aerosols from fluid drops [6]. Later, in 1882, Lord Rayleigh determined the amount of charge needed to overcome a surface tension of a drop [7]. Cooley patented electrospinning in 1902 and defined it as a device for electrically distributing fluids [8]. Anton Formhals developed preparation methods and published several patents from 1934 to 1944 [9]. Sir Geoffrey Ingram Taylor made a significant contribution to the theoretical understanding of electrospinning technology between 1964 to 1969 AD. He defined the characteristic droplet shape, which is commonly known as the ‘Taylor cone’ [10]. Despite these early discoveries, up until the 1990s, the process was largely forgotten. In the late 1990s, Ranker popularized electrospinning to explore the structural morphology of a wide range of polymeric nanofibers [4,11,12]. Since then, creating nanofibers via the electrospinning method has gained popularity.

**Figure 1:**
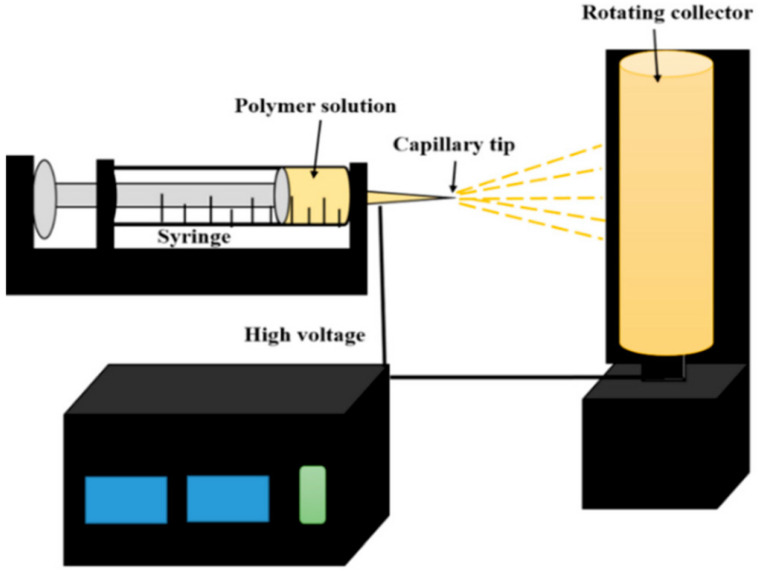
Schematic representation of a typical electrospinning setup [13].

A general electrospinning setup consists of four primary components: a high-voltage power supply, a capillary tube, a spinneret, and a grounded collector (Figure 1) [13]. In a typical electrospinning setup, a high voltage is applied to the polymeric solution, which electrifies a liquid drop to create a jet. The jet produces a single thread of fiber upon elongation and stretching, which is subsequently deposited onto a grounded collector. The electrospinning process is influenced by several parameters such as polymer concentration, solution viscosity, flow rate, electric field intensity, the distance from the tip to the collector, and air humidity. To obtain the required morphology of nanofibers, it is necessary to optimize the aforementioned parameters because they do not each work independently during electrospinning [2,13].

With the increasing popularity of nanofibers, electrospinning technology has been employed extensively. So far, more than 200 polymers have been electrospun into the nanofiber form [13,14]. The outstanding characteristics of electrospun nanofibers can be summarized below [11,13,15]:

(i) Electrospun nanofibers have a diameter ranging from nano to micro scale.
(ii) Electrospun nanofibers possess high porosity.
(iii) The fibers produced by the electrospinning technique have a large aspect ratio and a high surface-to-volume ratio.
(iv) Electrospun nanofibers possess superior mechanical properties and flexibility.
(v) The electrospinning process enables the production of nano/microfibers with an infinite number of chemical compositions.
(vi) Various types of morphology can be prepared by modifying the spinneret.

## 2. Biomedical Applications of Electrospun Nanofibers

**Figure 2:**
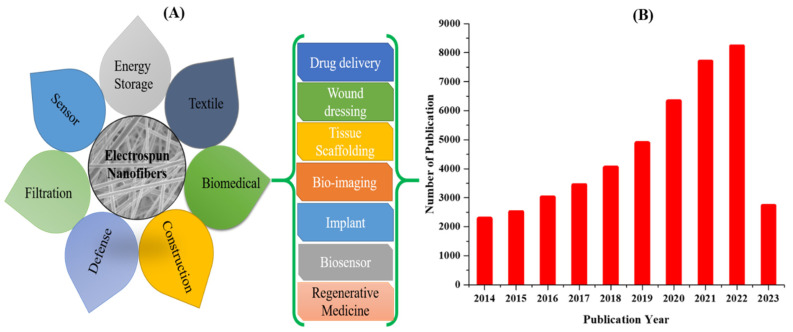
Various applications of electrospun nanofibers (**A**) and a graph showing the total relevant published articles on electrospun nanofibers for biomedical applications-related research from 2014 to April 2023 (**B**). The data were obtained by using the keywords ‘electrospun’ and ‘biomedical’ from Dimensions as of 10 April 2023.

Due to the aforementioned characteristics of electrospun nanofibers, they can be applied for numerous applications such as catalysis, energy storage, environmental remediation, filter media, sensor, textile, protective clothing, biomedicals, fire retardant, etc. [11,16,17,18,19,20,21,22,23,24,25,26,27,28,29]. Most importantly, the electrospun nanofibers can mimic the structure of the extracellular membranes (ECMs) [26,30,31]. The porous architecture of the electrospun nanofiber scaffolds is critical in cell survival, proliferation, and secretion of ESM [32]. Good pore connectivity allows the effective transport of nutrients, oxygen, and metabolic waste products to and from cells [33,34]. The biocompatibility and biodegradability provide a good environment for cell adherence, differentiation, and proliferation, thereby widening the applications of electrospun nanofibers in biomedical fields such as wound dressing [30,35], tissue scaffolds [36], drug delivery [13], cosmetics [37], implants [38], biosensor [39,40], antibacterial agent [16,41], etc. A schematic diagram showing some potential biomedical applications of electrospun nanofibers is given in Figure 2A. Figure 2B presents a survey of the total publications related to biomedical applications of electrospun nanofibers between 2014 to April 2023. (The quantitative data were derived from the Dimensions database).

In order to widen the applications of electrospun nanofibers in the biomedical field, a new form called ‘cell electrospinning’ (C-ES) was developed by Townesend-Nicholson et al. in 2006 [42]. The authors encapsulated live cells (1321N1) into polydimethylsiloxane nanofibers by coaxial electrospinning strategy. C-ES allows the encapsulation of whole microorganisms, such as bacteria, fungi, yeast, and viruses, as well as spores [43,44,45]. The nanofibers act as a cell reservoir and provide long-term reusability to the cells without hampering their activities [46,47,48].

### Electrospun Nanofibers for Dura Mater Repair

The brain is surrounded by three protective meningeal layers (pia mater, arachnoid mater, and dura mater). The dura mater is the outermost layer that surrounds and protects the brain and spinal cord [49]. It is made up of fibrous tissue, including collagenic and elastic fibers, and functions as a barrier to contain cerebrospinal fluid (CSF) within the cranial cavity. The dura mater can be divided into two layers [50,51]. The dense inner layer of the dura mater is the meningeal dura, which serves as a barrier by preventing the leakage of CSF and adhesion of brain tissue, guarding against superficial cerebral infection, and maintaining intracranial pressure. The outer layer of the dura mater is the periosteal dura, which often adheres to the cranial bone. Its porous surface is advantageous to autologous dura mater and bone regeneration. The dura mater can be damaged by trauma to the brain and spinal cord, tumor invasion, surgical resection, and congenital malformations, which can lead to several complications, including CSF leakage, infections, meningitis, and epilepsy [50,52]. Accessing the underlying nervous tissue during neuro and spinal surgery may cause damage or defect on the spinal dura mater. Therefore, artificial dura substitutes are required after a neurosurgical operation to prevent leakage, infection, and adhesion or to reduce the risk of dura mater defect. Due to the increasing cases of dural damage, dura repair has become one of the major medical problems in modern society. The dural tissue is non-regenerative; therefore, the substitute material should quickly seal leaks and promote the formation of surrounding tissues that are close to the defect [53]. During the last decade, various substitutes, including autograft, allograft, xenograft, and synthetic materials, have been used for the treatment of dural damage [54,55,56,57,58]. Due to nontoxicity and their rapid integration into native tissue, autografts are commonly preferred to repair dural defects during surgery; however, they have limited availability and require an additional incision at the graft harvesting site [56,59]. Although allografts are readily available, their use has become less favorable due to the high risk of transmission of the Creutzfeldt–Jakob disease (CJD) [60,61,62,63]. Collagen substitutes (xenografts) derived from bovine or porcine sources can also be used as dural substitutes; however, they may bring the risk of complications associated with infection and immunorejection [55,64]. In addition, the poor mechanical properties and rapid degradation of collagen hinder their application in dural repair [59,65]. Therefore, a promising alternative to the above substitute is urgently needed. Recently, dural substitutes based on synthetic materials have been studied as an alternative to reconstructing dural defects [66,67]. However, most of the substitutes fail to mimic the physiological structure of the dura mater and hence are less effective. To obtain the desired results, an ideal dura substitute should satisfy the following conditions [67,68]:

- It must be biocompatible.
- It should mimic the physiochemical structure of the dura mater.
- It should adequately restore the continuity of the dura mater and prevent the leakage of CSF.
- It should lower the chances of infection.
- The mechanical properties of the substrate should facilitate the suturing.
- It should enhance tissue regeneration.
- It should promote the adhesion and migration of dural fibroblasts.
- It should minimize local tissue inflammation.
- It should not induce adverse reactions.
- It should be safe, inexpensive, and easy to handle.

The US Food and Drug Administration (FDA) (U.S. Food and Drug Administration, 2000) and The National Medical Products Administration of China (NMPA, 2020) suggest that the ideal dural substitute should include the following attributes [51]:

- Biocompatibility without inducing an immune or inflammatory response.
- Controlled risks of infection.
- Appropriate mechanical properties, which are resistant to tearing
- Anti-leakage of CSF.

In the last decade, new artificial polymeric dural substitutes have been developed, some of which are in clinical trials. Nanoabsorbable synthetic polymers such as silicon and expanded polytetrafluoroethylene have been used for the dural implant; however, it may cause serious complications such as induction of granulation tissue formation due to long-term foreign body reaction [69,70]. Natural polymers such as collagen, fibrin, alginate, and cellulose have also been applied for various biomedical applications, including dural implants; however, these polymers are not free from the potential risk of infection [55,71,72,73]. As a result, various synthetic polymers such as poly lactic acid (PLA), polyurethane (PU), polyethylene glycol (PEG), poly-E-caprolactone (PCL), poly (3-hydroxybutyrate-co-3 hydroxyvalerate (PHBV), polyglycolic acid (PGA), and etc. have been applied for synthesizing dural substitutes [52,66,67,74,75,76,77,78,79,80]. Although synthetic polymers possess sufficient mechanical properties, most of them are rejected due to local tissue reactions, excessive scar formation, meningitic symptoms, etc. On the other hand, the natural polymers-based dural substitutes have insufficient mechanical strength, which may cause CSF leakage. So far, various dural substitutes have been tested in the form of hydrogels, films, meshes, and glues [80,81,82]. The isotropic surface properties of these substitutes are not well suited for cell attachment and proliferation, which results in poor performance of the material [74]. Among them, various tough hydrogels have been prepared, which possess a good load-bearing capacity to fit the desired mechanical properties for dural implants [53,82,83,84,85,86,87]. However, the poor biocompatibility and degradability of these synthetic polymers limit their application as a dural substitute. Furthermore, it is challenging to design hydrogels with desired networks that can mimic the dura mater morphology. Therefore, there is an urgent need to develop dural substitutes with good mechanical properties and biocompatibility. In this regard, electrospun polymeric nanofibers have been accepted as an excellent alternative material due to their exciting features, including a high surface area to volume ratio, porosity, flexibility, good mechanical strength, mimicking the ECM morphology, ease of surface modification [13,88]. The three-dimensional structure of electrospun polymeric nanofibers is similar to the fibrous structure of dura mater [89,90]. The fibrous structure provides sufficient adhesion sites as well as offers mechanical support to the dura cell during the tissue repair process. The electrospun nanofibers scaffolds with various alignments of nanofibers have proven to have a superior capacity in fast recovery of dura mater [74,91]. In addition, the nanofibers can be made in a composite form, and various growth factors, drugs, or biologically active materials can be encapsulated into them easily [13]. Therefore, electrospun nanofiber membranes can be considered promising materials in dura substitutes. The development of electrospun nanofibers for dura mater regeneration is summarized below (Table 1).

It is of great significance to develop an artificial dura substitute that matches the intrinsic structure and mechanical properties of natural dura mater. Zwirner et al. [99] investigated the mechanical properties of human temporal dura mater and recorded the tensile strength of 7 ± 4 MPa. Ma et al., for the first time, synthesized a nanofiber membrane of poly(4-hydroxybutyrate) (P_4_HB) possessing good mechanical strength, flexibility, wettability, and biocompatibility via chemosynthesis and electrospinning methods [94]. The in vitro study showed fast cellular migration, adhesion, and proliferation of fibroblasts. The authors further studied the in vivo study in rats, which demonstrated excellent biocompatibility with proper biodegradation. As an onlay dural graft, the membranes prevented CSF leakage and regenerated dura tissue without any foreign body response.

**Figure 3:**
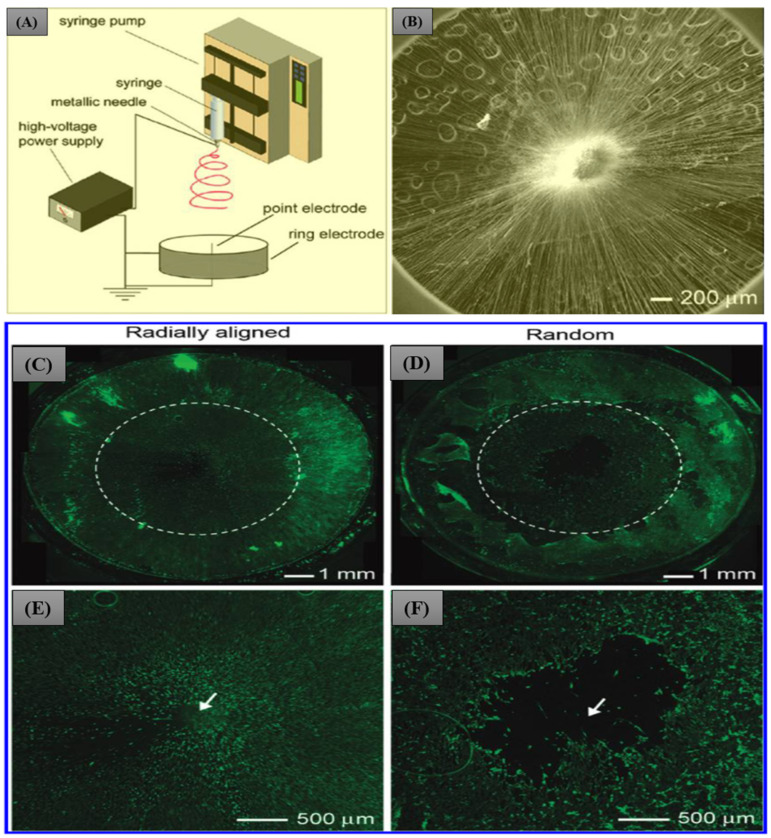
Schematic diagram showing the electrospinning setup for producing radially aligned nanofibers (**A**), SEM image of aligned fibers (**B**), fluorescence micrographs comparing the migration of cells after culturing the dura tissue on the aligned (**C**,**E**) and random fibers (**D**,**F**) for 4 days. The arrows in (**E**,**F**) represent the center of the scaffold. Reproduced with permission [74]. Copyright 2010, American Chemical Society.

Control of the fiber orientation is highly desirable to obtain increased complexity and performance [100]. Recently, aligned nanofibers have been extensively investigated in the biomedical field due to their significance in mimicking biological cells and controlling cell behavior [101,102]. The electrospun-aligned nanofibers resemble the ECM and hence provide an adequate growth environment for the cells [102]. In addition, the aligned nanofibers possessed enhanced mechanical properties, high surface area, and porosity [103,104]. Xie et al. [74] designed aligned PCL nanofibers by electrospun for the first time to study their possibility in dural substitute. PCL is an FDA-approved, semicrystalline polymer that can be degraded into non-toxic products. Due to this reason, PCL has been applied for various biomedical applications, including dura substitutes [105,106,107,108,109]. The authors utilized a collector composed of a central point electrode and a peripheral ring electrode to design the random fibers (Figure 3A,B). The scaffold presented migration of the cultured cells from the periphery to the center. As compared to the random orientation, the aligned nanofibers membrane induced faster cellular migration and population (Figure 3C,E), whereas the acellular region was observed at the center of the scaffold made of random nanofibers (Figure 3D,F).

**Figure 4:**
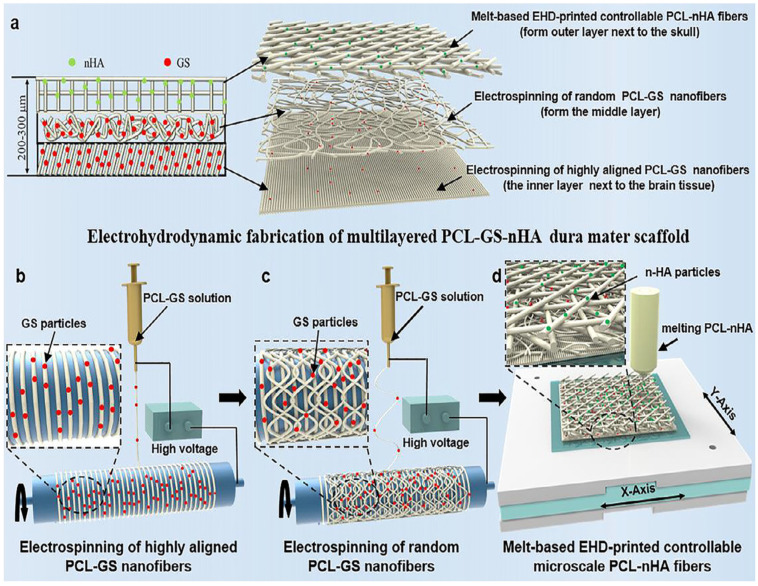
Schematic diagram showing the electrohydrodyamic fabrication of triple-layered micro/nano fibers of PCL-GS-nHA dura mater scaffold (**a**), electrospinning of aligned and random PCL-GS NFs ((**b**,**c**), respectively), and preparation of microscale PCL-nHA fibers by melt-based EHD printing (**d**) [49].

Integration of two or more materials in a composite hold great potential in dural repair [110]. The composite nanofibers from natural and synthetic polymers have shown desired structures and material properties for biomedical applications, including dural repair. Natural materials provide favorable biological properties, whereas synthetic materials provide mechanical strength [111,112]. In addition, the fabrication of a multilayered structure could mimic the microarchitecture and multiple functions of native dura mater. Su et al. [49] prepared a triple-layered dura mater substitute by electrospinning and melt-based electrohydrodynamic jetting techniques (Figure 4). They prepared highly aligned polycaprolactone (PCL) nanofibers loaded with gentamicin sulfate (GS) to mimic the aligned collagen fibers of the native dura mater. Randomly deposited PCL-GS fibers were used as a middle layer. The random fibers in the composite enhanced the mechanical properties of the scaffolds. The outer layer was made up of PCL microfibers containing nano-hydroxyapatite (nHA). The outer layer served as an effective layer for improving its integration with the native skull. The mechanical properties of the triple-layered scaffolds were found to be comparable with the natural porcine dura mater. In addition, the presence of GS and nHA brought antibacterial properties and significantly promoted osteogenic differentiation. Similarly, Wang et al. [52] fabricated a multilayer biomimetic scaffold using PLA, PCL, and collagen to promote dural repair. The inner layer was composed of PLA, which was helpful in reducing tissue adhesion. The middle layer was composed of PCL and PLA, which provided mechanical strength and a watertight seal. The outer layer was made up of collagen and PLA to enhance cell attachment and proliferation. The scaffold was implanted in rabbits and the results showed that the scaffold had sufficient mechanical strength and biochemical properties to enhance dural repair. Kyale Kurpinski and Shyam Patel [92] prepared a nanofiber dural substitute by electrospinning, which was composed of poly(dl-lactide-co-e-caprolactone) (PLCL), poly(propylene glycol) (PPG), and sodium acetate. The prepared nanofiber membrane was aimed to enhance dural healing via biomimetic nanoarchitecture and support both onlaid and sutured implantation. They investigated the morphological, mechanical, and handling properties of the bilayer nanofibrous dural substitute and evaluated the biological performance of the dural substitute in a canine duraplasty model. When implanted, the nanofibrous graft prevented leaks and brain tissue adhesions, and encourages dura mater regrowth, performing comparably to the collagen matrix. Both in vitro fibroblast orientation and in vivo dural healing was enhanced by the aligned nanofibers. Chuan et al. [89] prepared stereocomplexed composite nanofiber membranes using PLA and poly (D-lactic acid) PDLA-grafter tetracalcium phosphate (TTCP) by the electrospinning method. The tensile strength and elongation break of the nanofiber membrane were recorded as 6.46 ± 0.07 MPa and 111.2 ± 4.7%, respectively, which are close to human dura mater. They investigated the in vitro cytotoxicity and proliferation of bone marrow stem cells on the nanofiber membrane, and the obtained results indicated neuron compatibility suggesting its potential application as a dura substitute. Zhao et al. [96] electrospun PLGA, tetramethylpyrazine, and chitosan into a nanofiber membrane with antifibrotic and neuroprotective effects by coaxial electrospinning technique for artificial dural substitute. The prepared membrane inhibited the excessive proliferation of fibroblasts, exerted anti-adhesion effects, and inhibited the formation of scar tissue.

Deng et al. [113] designed PLLA/gelatin biomimetic substitute and studied the biological characteristic in vitro and tissue regeneration in vivo. The results demonstrated that the composite substrate could promote the growth and regeneration of dural cells. In addition, it effectively prevented CSF leakage and local infections. The nanofiber orientation affects the morphology, electrical, optical, and mechanical properties of the dural substrate leading to changes in cell behavior. The effects of fiber orientation of the scaffolds on the expression/proliferation of various types of cells have already been reported in the literature [74,114,115,116,117,118].

**Figure 5:**
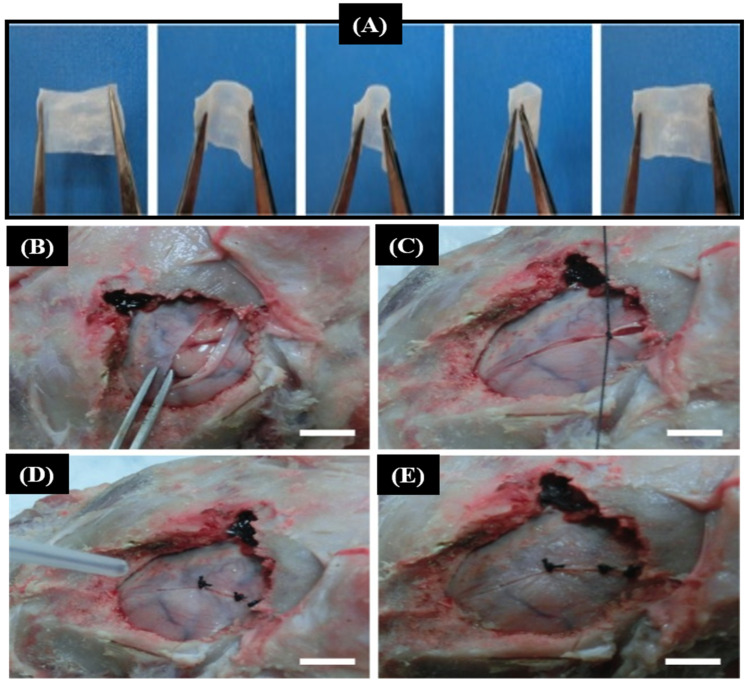
Optical images showing the flexibility of the NOCA membrane (**A**). Optical images for the in vivo simulation experiment. (**B**) A defect of 7 cm in length. (**C**) The dura was primarily sutured to avert rupture. (**D**) The NOCA fibers on the defect. (**E**) Repaired defect by the NOCA membrane [93].

CSF leakage is one of the major postoperative complications which requires surgical intervention. Recently, various biocompatible polymers have been proposed as tissue adhesives for closing surgical defects and incisions [119,120]. In this regard, Lv et al. [93], for the first time, proposed an in situ precise electrospinning of *n*-octyl-2-cyanoacrylate (NOCA), a medical glue, into fiber membrane for treating dural defects. The in vitro and in vivo experiments were carried out on egg membranes and goat meninges, respectively. The results showed that the NOCA membranes had properties of high strength, good flexibility, and waterproofness without leakage (Figure 5A). Most importantly, the in vivo experiment demonstrated that this flexible membrane not only rapidly sutures dural defects but also avoids tissue adhesion (Figure 5B–E). Adhesion between the scaffold membrane and the temporal muscle is one of the limiting factors of artificial scaffolds in dura repair. To address this issue, Shi et al. [98] prepared PCL/gelatin nanofiber membrane via an electrospinning technique. The composite membrane had high tensile strength, good biocompatibility, and a long-term in vivo degradation rate. The anti-adhesion property was checked in rabbit skull, and it showed an efficient anti-adhesion barrier.

In order to avoid cerebrospinal fluid leakage during the neuro- and spinal surgery, Yu et al. [77] developed a package that includes two layers of electrospun membranes, the poly(lactide-co-glycolide) (PLGA) with oriented microstructure as the inner layer and chitosan-coated electrode nonwoven PlGA membrane as an outer layer. The inner layer served as a substrate to anchor dermal fibroblast, whereas the outer layer enhanced the mechanical properties of the substrate. The prepared scaffold was applied to goats with dural defects in the lumbar. The seamless and quick sealing of the defect area with regeneration in the defects was realized.

Although major advances have been made in the fabrication of nanofibers membranes for dural repair, these scaffolds do not accurately capture all of the biological functions of the dura mater. Loading neurotropic factors into the electrospun nanofibers regulate and promote the differentiation, growth, and survival of nerve cells [68,100,121,122,123,124]. Fibroblast growth factors induce DNA synthesis and cell migration and promote wound healing [125]. Table 2 summarizes some studies on neurotropical factor-loaded electrospun nanofibers for dural implants. Mohtaram et al. [126] synthesized glial cell-derived neurotrophic factors (GDNF)-encapsulated random and aligned nanofibers of PCL that could serve as an artificial dura. They performed a release study to determine the kinetics of GDNP release from the scaffold over 30 days. The controlled release of GDNF promoted the survival of neurons during neurosurgical procedures, and hence, it could be used to treat CNS disorders caused by membrane disruption. For effective spinal cord regeneration, inhibitory factors for axon growth and appropriate axon guidance in the lesion region are important. Zhu et al. [78] developed a nanofiber scaffold that not only guides axon growth but also releases drugs to promote the regeneration of spinal cord tissue. They prepared composite nanofibers of PLLA and PLGA with a two-layer structure: aligned and random fibers in the inner and outer layers, respectively. Further, to test the therapeutic effect, they immobilized rolipram in the nanofibers and applied rats. They observed that the drug-loaded scaffold not only promotes axon growth and angiogenesis but also suppresses glial scar formation. Shi et al. [127] designed a drug-loaded double-layered electrospun nanofiber membrane to prevent epidural adhesion. Both layers were made up of polycaprolactone and chitosan in different weight ratios. The bottom layer, which contracts the dura, was loaded with meloxicam to prevent inflammation, whereas the top layer, contracting to the fibrous tissue, was loaded with mitomycin-C to inhibit the synthesis of DNA and collagen. This study showed the drug release from the membrane for 12 days. In vitro studies confirmed that the drug-loaded membranes were non-cytotoxic and could inhibit fibroblast proliferation. Dural adhesion and scar tissue formation are the common problems associated with dural grafts. To solve these problems, Zhao et al. [96] fabricated a nanofiber membrane composed of PLGA, tetramethylpyrazine, and chitosan. The composite nanofiber membrane exhibited excellent biocompatibility and adequate mechanical properties and could play a neuroprotective role. It exerted anti-adhesion effects and inhibited the formation of scar tissue. Introducing multifunctional dura substitutes may address the multiple problems associated with artificial dura mater materials. In this regard, Liao et al. [128] proposed a multifunctional dura substitute composed of gel and electrospun nanofibers. They used PLLA, chitosan, gelatin, and small intestinal submucosa (SIS) to prepare a triple-layered composite scaffold. The composite showed multiple functions, such as leakage blockade, adhesion prevention, antibacterial performance, and dura reconstruction potential. Sanpakitwattana et al. [129] incorporated cefazolin into an oxidized regenerated cellulose/polycaprolactone composite membrane to introduce antibacterial properties to the dura substitute. The composite membrane possessed physical and mechanical properties in the range of natural dura mater. The membrane exhibited a monophasic burst release of cefazolin; however, the antibacterial activity was sustained for 4 days.

## 3. Conclusions, Challenges, and Future Perspectives

The dura mater is the outermost layer that surrounds and protects the brain. It plays a key role in keeping stable intracranial pressure. Various factors such as trauma, inflammation, tumor invasion, surgical resection, and congenital malformations can cause dura mater defects, and various types of dural substitutes have been used to deal with these defects. Recently, Electrospinning has gained intensive attention as a versatile technique to prepare organic/inorganic nanofibers membranes for various applications, including biomedical. In the last decade, impressive progress has been made in applying electrospun nanofibers in dura mater regeneration. Several biocompatible polymers and their composites have been prepared with various fiber orientations (random, aligned, bi-layer, tri-layer, etc.) and investigated for dural implants.

Although electrospun nanofiber membranes can serve as an effective scaffold for dura repair, there are a few limitations that hinder their application in this field. For example, the dura substitute should mimic the intrinsic structure of the human dura mater. It is challenging to fabricate an electrospun nanofiber membrane with precise dimensions and morphology. Poor mechanical properties and rapid degradation, particularly in the case of natural polymers, are also a challenge to overcome. Authors have attempted to address this issue by fabricating composite nanofibers from natural and synthetic polymers. However, it is difficult to obtain properly blended natural-synthetic composite nanofibers due to the poor miscibility of the component polymers. In some cases, the solvents may alarm additional issues such as miscibility, biocompatibility, pungent smell, etc. Aligned nanofiber membranes have been developed to enhance mechanical properties and cell growth. However, it may need a modified electrospinning setup. Another concern is the adhesion formation between the scaffold membrane and overlying tissue. In order to obtain proper and fast growth of nerve cells, neurotropic factors-incorporated nanofibers membranes have been developed. The potential side effects and achieving controlled release are the major concerns. Therefore, several technical and pharmacological issues should be addressed prior to exploiting their potential. Safety is another issue in electrospinning as high voltage is used during its operation.

It is evident from the literature that most of the studies are at the lab scales, and further studies are needed for their clinical applications. It means that there is still a need for an optimized dural substitute with safety, proper mechanical properties, and low cost. By optimizing various properties of nanofibers membranes in terms of fiber orientation, morphology, porosity, drug release, mechanical properties, and, most importantly, biocompatibility, the product can fulfill specific requirements. With the rapid development of materials science and ongoing research on the dura substitutes, it can be hoped that new implant materials will be developed soon, which can overcome all the limitations of the currently available products.

## Figures and Tables

**Table 1:** Various electrospun nanofibers for dura mater regeneration.

SN	Materials	Results	Ref.
1	PLCL, PPG and sodium acetate	-Bilayer dural substitute having aligned nanofibers on one side and random nanofibers on the other. -Significantly high strength and durability compared to commercially available collagen matrix. -In vitro fibroblast and in vivo dural healing were enhanced by the aligned nanofibers.	[92]
2	PCL	-Proposed a new setup for producing aligned nanofibers by electrospinning. -Migration of cells from periphery to the center. -Potentially allowing for fast regeneration and formation of neodura.	[74]
3	poly(lactide-co-glycolide)	-A package with two layers of electrospun membranes, dermal fibroblast and mussel adhesive protein and used in a goat. -Electrospun PLGA and chitosan coated PLGA membranes were used as inner and outer membranes, respectively. -Guided tissue growth and regeneration in the defects were observed.	[77]
4	PLA-PCL-Collagen	-Three-layered scaffold (PLA-PCL-collagen serve as inner to outer layer). -Sufficient mechanical strength and biocompatible.	[52]
5	*n*-octyl-2-cyanoacrylate/poly(methyl methacrylate)	-High compactness and flexibility -Experiments on egg membranes and goat meninges showed rapid and effective recovery in dural defect.	[93]
6	PCL/GS/nHA	-Biomimetic triple-layered membrane. -Comparable mechanical properties to natural dura mater. -Good biocompatibility with anti-infection properties.	[49]
7	PLLA, poly(D-lactic acid)-grafted tetracalcium phosphate	-Tensile strength close to human dura mater. -Non-toxic and neuron compatible.	[89]
8	poly(4-hydroxybutyrate) (P_4_HB)	-Good mechanical properties that match the natural dura mater. -Induces fast cellular migration, adhesion, and proliferation of fibroblasts in vitro. -Implantation in rats demonstrates excellent biocompatibility of the P_4_HB membrane with proper biodegradation behaviors.	[94]
9	*n*-octyl- 2-cyanoacrylate (NOCA)	-Studied for in situ dural closures after neurosurgery. -The fiber membrane showed significantly higher sealing capabilities of defects in human dura.	[95]
10	PLGA/CS	-Can inhibit the excessive proliferation of fibroblasts, as well as provide a sustained -Protective effect on the human neuroblastoma (SH-SY5Y) cells treated with oxygen–glucose deprivation/reperfusion	[96]
11	Bacterial cellulose	-First study on using bacterial cellulose for rabbit dural defect. -Good biocompatibility in vitro and in vivo. -Implantation study showed no relevant complications. -Mild local inflammatory reaction detected.	[97]
12	PCL/gelatin	-The mechanical strength was increase with the PCL content whereas biocompatibility was increased with gelatin content. -Subcutaneous implantation in rabbit for 6 months exhibited adjustable biodegradable behavior.	[98]

Polycaprolactone (PCL), poly(dl-lactide-co-e-caprolactone) (PLCL), poly(propylene glycol) (PPG), polylactic acid (PLA), poly-L-lactic acid (PLLA), poly (lactic-co-glycolic acid) (PLGA), chitosan (CS), poly(4-hydroxybutyrate) (P_4_HB).

**Table 2:** Various neurotropic factors loaded electrospun nanofibers for dura repair.

S.N.	Materials	Results	Ref.
1	mitomycin-C and meloxicam loaded PCL/CS fibers	-Drug-loaded double-layered membrane. -Bottom layer is loaded with meloxicam to prevent dural inflammation. -Top layer is loaded with mitomycin-C to inhibit the DNA synthesis. -Both drugs were released for 12 days. -It prevented the epidural adhesion formation.	[127]
2	tetramethylpyrazine/PLGA/CS NFs	-Excellent biocompatibility, adequate mechanical properties and good antifibrotic effects. -Inhibits excessive proliferation of fibroblasts. -Brought anti-adhesive effects and inhibited the formation of scar tissue.	[96]
3	icariin-loaded PCL/gelatin NFs	-Prevent fibroblast adhesion and proliferation. -In vivo studies with rabbit laminectomy models showed the release of ICA in a controlled and sustained manner.	[130]
4	PCL/hyaluronic acid methacryloyl (HAMA)/IGF-1 NFs	-Long-term release of growth factor. -hydrophobic membrane with good mechanical properties. -Improve the microenvironment of neurite growth and promote the survival of neural cells.	[107]
5	PLGA-Graft-PVP/PC NFs	-No cytotoxic effect -Safe and effective physical barrier for preventing epidural adhesion.	[131]
6	PCL-BSA-GDNF	-Random and aligned fibers -GDNF release -Support the culture and differentiation of hiPSC-derived neural progenitors	[126]
7	rolipram/PLLA/PLGA NFs	-Aligned nanofibers in inner and random fiber in outer layer. -Effective in guiding axon growth and angiogenesis and releasing drug.	[78]
8	SIS loaded PLLA/CS/gelatin NFs	-Combination of hydrogel and electrospun nanofibers with triple-layered structure. -SIS helped to improve bioactivity. -Good dura reconstruction potential with interesting features such as leakage blockade, adhesion prevention, and antibacterial properties.	[128]
9	cefazolin loaded ORC/PCL NFs	-Initial burst release one the first day followed by constant and slow release of cefazolin. -Antibacterial activity for 4 days.	[129]
10	lidocaine embedded PLGA NFs	-Biodegradable nanofiber membrane for epidural analgesia. -Sustainable release of lidocaine for more than two weeks.	[132]

Glial cell-derived neurotrophic factor (GDNF), bovine serum albumin (BSA), human induced pluripotent stem cells (hiPSCs), insulin-like growth factor 1 (IGF-1), polyvinylpyrrolidone (PVP), small intestine submucosa (SIS), oxidized regenerated cellulose (ORC).

## Data Availability

Not applicable.
